# Severe Macular Ischemia Is Associated with a Poor Visual Prognosis and Serious Complications in Eyes with Central Retinal Vein Occlusion

**DOI:** 10.3390/jcm12216710

**Published:** 2023-10-24

**Authors:** Ryo Kurobe, Yoshio Hirano, Takaaki Yuguchi, Norihiro Suzuki, Tsutomu Yasukawa

**Affiliations:** Department of Ophthalmology & Visual Science, Nagoya City University Graduate School of Medical Sciences, 1-Kawasumi, Mizuho-cho, Mizuho-ku, Nagoya 467-8601, Japan; monokurokuroboo1025@gmail.com (R.K.); n.suzuki11@gmail.com (N.S.); yasukawa@med.nagoya-cu.ac.jp (T.Y.)

**Keywords:** macular ischemia, central retinal vein occlusion, visual prognosis

## Abstract

Purpose: This study aims to investigate the factors influencing post-treatment visual acuity (VA) in patients with central retinal vein occlusion (CRVO) with macular edema (ME). Methods: The subjects of this study were patients who visited our clinic from May 2013 to July 2019 and who could be followed up with for at least 12 months. Cases with hemi CRVO were excluded from this study. Factors considered in the evaluation of visual prognosis at the 12 months included initial best-corrected VA, central subfoveal thickness, CRVO subtype (nonischemic, ischemic, or converted from nonischemic to ischemic), time taken for the first treatment, number of anti-vascular endothelial growth factor agent injections, structural changes in the inner and outer retinal layers, and the presence of macular ischemia in a multiple regression analysis. Results: There were 41 patients with 41 eyes, 27 males and 14 females. The mean age of the patients was 70.5 ± 12.2 (mean ± standard deviation) years. The mean VA was 0.544 ± 0.576, 0.456 ± 0.568, and 0.586 ± 0.665 at the initial visit, 12 months later, and time of last observation, respectively. There were no significant differences in VAs observed between the baseline, month 12, and final visit. Multiple regression analysis revealed that the external limiting membrane score at month 12 (*p* = 0.030), the VA at initial visit (*p* < 0.001), and the presence of severe macular ischemia (*p* < 0.001) were the key factors associated with VA at month 12. Moreover, severe macular ischemia was identified as the only factor affecting decimal VA less than 20/200 at the last observation (*p* = 0.0092). Conclusions: Severe macular ischemia is strongly linked to a poor visual prognosis in patients with ME associated with CRVO.

## 1. Introduction

The main cause of vision loss in retinal vein occlusion (RVO) is macular edema (ME) [1,2]. Anti-vascular endothelial growth factor (VEGF) therapy has dramatically changed the treatment of various ME-associated retinal choroidal diseases [3,4,5], and in eyes with RVO, as shown by previous large clinical trials [5,6,7,8,9,10,11,12,13,14,15,16], anti-VEGF therapy has provided good visual acuity (VA) improvement in both branch RVO (BRVO) and central RVO (CRVO). In daily practice, however, the VA prognosis for BRVO is relatively good, whereas in cases with CRVO, we often encounter cases in which VA does not improve even after the ME resolves, or in which there are serious complications and a poor prognosis for VA. The discrepancy between large-scale clinical trials and daily clinical practice can be attributed to the stringent entry criteria of these trials, which often exclude cases with poor initial vision or significant retinal ischemia and which as a result fail to fully replicate real-world scenarios. In daily practice, there are a wide variety of cases in which there is a poor VA of below 0.05 decimal, and in which there is an already developed neovascular glaucoma (NVG). Nagasato et al. [17] conducted an analysis encompassing a population closely resembling real clinical practice, including patients with poor initial vision. Their findings indicate that the visual prognosis of CRVO is dichotomous, i.e., that the effect of CRVO is clearly different between those with a favorable prognosis and those with a poor prognosis.

Numerous studies investigating the visual prognosis in CRVO have highlighted how the significances of initial VA and age are crucial prognostic indicators [18,19,20]. Other factors, such as the status of the outer retinal layers, including the external limiting membrane (ELM [21]) and the ellipsoid zone (EZ [22]), have been identified as influencing VA outcomes. These findings are partly attributed to the advancements in optical coherence tomography (OCT) technology, enabling the detailed assessment of the retinal structure.

Moreover, several ischemic biomarkers detected by OCT, such as the disorganization of retinal inner layers (DRIL) [23] and the prominent middle limiting membrane (p-MLM) [24], along with the quantification of vascular density by OCT angiography (OCTA), can be utilized to evaluate macular ischemia [25], which has been linked to visual outcomes. In addition, CRVO is classified into ischemic and nonischemic types based on the status of retinal perfusion, with the ischemic type generally associated with a poorer prognosis compared with the nonischemic type [26]. This classification is related to the presence of severe macular ischemia and the development of severe complications, consequently leading to a compromised visual prognosis [26].

In addition, the occurrence of serious complications such as vitreous hemorrhage (VH) and NVG can result in substantial vision loss in patients with CRVO. Anticipating the onset of these complications beforehand is crucial in facilitating timely intervention following the onset of CRVO, thereby emphasizing the critical importance of effective CRVO management. Consequently, the objective of this study was to identify the factors contributing to the visual prognosis of CRVO and the likelihood of encountering severe complications.

## 2. Methods

STUDY DESIGN AND SETTING: This was a retrospective, observational, consecutive case series conducted in an institutional setting. The study protocol was approved by the Institutional Review Board of Nagoya City University Graduate School of Medical Sciences (No. 60-20-0068). All patients provided written informed consent for participation in the study. The described research methods and analysis adhered to the tenets of the Declaration of Helsinki.

Patients who visited Nagoya City University Hospital from May 2013 through July 2019 were subjected in this study. The inclusion criteria included CRVO patients with ME who were followed up with for more than 12 months and who underwent OCT, and OCTA or fluorescein angiography (FA) at baseline and 6 months after initial visit. The exclusion criteria included eyes with other retinal and/or choroidal diseases or hemi-CRVO, patients who underwent a follow-up period that was shorter than 12 months, patients whose images were too poor quality to be analyzed because of eye movement, media opacities due to corneal diseases and/or cataract, eyes with thick retinal hemorrhage, patients with severe ME, patients with other retinal disease, and patients with high myopia greater than −6.0 diopters. Infected eyes such as with patients suffering endophthalmitis or other bacterial infections after injection of anti-VEGF agents were also excluded from this study.

OBSERVATIONAL PROCEDURE: All patients underwent a complete ophthalmic examination, including measurement of best-corrected VA (BCVA) and intraocular pressure (IOP), indirect ophthalmoscopy, fundus photography (Optos California, Tokyo, Japan, Nikon Healthcare Japan, Inc., Tokyo, Japan, and TRC-50AX, Topcon, Tokyo, Japan), OCT (Cirrus HD-OCT model 6000, Carl Zeiss Meditec, Dublin, CA, USA; AVANTI OCT, AngioVue, Optovue Inc., Fremont, CA, USA), OCTA (RTVue XR Avanti, AngioVue, Optovue Inc., Fremont, CA, USA), and/or FA. FA was performed using ultra-widefield laser ophthalmoscope (Optos California) and/or confocal scanning laser ophthalmoscopy (Heidelberg Retina Angiograph 2 (HRA2), Heidelberg Engineering, Heidelberg, Germany). In all eyes, BCVA measurement, indirect ophthalmoscopy, and OCT were examined every month after the initial visit (baseline). OCTA and/or FA also was performed in all eyes at baseline and 6 months after initial visit.

ASSESSMENTS: All values were expressed as the mean ± standard deviation (SD). BCVA was measured using a Landolt C chart and converted to the LogMAR for statistical analysis. In accordance with a previous report [27], LogMAR VA for finger counting, hand motion, light perception, and no-light perception was set as 2.6, 2.7, 2.8, and 2.9, respectively. Central subfoveal thickness (CST) was measured on the OCT system automatically. The mean LogMAR VAs and the mean CSTs were compared between baseline, 12 months after the initial visit, and the final visit. Eyes with larger non-perfusion areas (NPAs) than 10-disc areas were defined as ischemic types and the others as nonischemic. Subtype determination and conversion from nonischemic to ischemic types were undertaken with FA and/or OCTA images from two retinal specialists (R.K. and N.S.) in a masked fashion. If their evaluations differed, a masked third investigator (Y.H.) joined in the discussion to determine the type. In a similar way, DRIL scores on OCT vertical and horizontal section images were graded from 0 to 3 as previously reported [28] and their mean was calculated. P-MLM is a hyperreflective line located inside the outer retinal layer, which corresponds to the outermost layer nourished by retinal vessels, and its presence indicates acute retinal ischemia [24]. The presence or absence of p-MLM was determined based on the OCT images. Regarding ELM and EZ, the scores were graded from 0 to 3 based on OCT vertical and horizontal section images [29], respectively, and the mean of the scores was calculated. Macular ischemia was determined on OCT or FA images and was evaluated on the retinal superficial layer. For evaluation by OCTA images, a 3 × 3 mm OCTA image centered on the fovea was taken, and the vascular density of the retinal superficial layer within that area was calculated by the built-in software. Then, using the data, macular ischemia was graded as follows: vascular density of 40% or greater was defined as no macular ischemia (Grade 0), vascular density of 35% to 40% as mild macular ischemia (Grade 1), and vascular density of 35% or less as severe macular ischemia (Grade 2) (Table 1). For evaluation by FA, early treatment diabetic retinopathy study grid images were evaluated and one or more areas of NPAs in the central, outer, and inner regions were defined as having macular ischemia. With some modifications to the previous report [14], mild macular ischemia was defined as less than half of the subfield corresponding to the NPA, and severe macular ischemia as half or more of the subfield corresponding to the NPA.

TREATMENTS: ME was treated with intravitreal injection of anti-VEGF agents [either 0.5 mg/0.05 mL ranibizumab (IVR) (Lucentis^®^, Genentech Inc., South San Francisco, CA, USA) or 2.0 mg/0.05 mL aflibercept (IVA) (Eylea^®^, Regeneron Pharmaceuticals Inc., Tarrytown, NY, USA)]. For economic reasons and/or systemic complications, triamcinolone acetonide (TA) (MaQaid^®^, WAKAMOTO PHARMACEUTICAL Co., Ltd., Tokyo, Japan) was injected into either the sub-Tenon’s capsule (STTA) or the vitreous cavity (IVTA). Additional treatment for ME was performed when the CST exceeded 300 μm in one plus pro re nata (PRN) regimen over 12 months with a monthly visit. Pan-retinal photocoagulation (PRP) was performed for the widespread NPAs that were larger than a 10-disc area and/or for retinal neovascularization (NV). Vitrectomy was performed for persistent VH, or NVG with unclosed angles, and a laser with a scleral indentation was applied firmly to the peripheral retina. Glaucoma surgery was performed for NVG with poorly controlled IOP.

STATISTICS: Statistical analysis was performed using Easy R software Ver. 1.40 (Jichi Medical University Saitama Medical Center, Saitama, Japan), which is a graphical user interface for R (The R Foundation for Statistical Computing, Vienna, Austria). More precisely, it is a modified version of R commander designed to add statistical functions that are frequently used in biostatistics [30], with *p* < 0.05 set to represent significant difference. One-way and repeated-measures analyses of variance with the Bonferroni correction were used to estimate the continuous outcome measures: LogMAR VA and CST at baseline, month 12, and final visit. Welch two sampled *t*-test was used to compare baseline LogMAR VA and baseline CST between samples that were or were not nonischemic, and between samples that showed severe macular ischemia or not. Univariate analysis was performed to detect factors predictive of the LogMAR VA at month 12 and risk factors for a final decimal VA of less than 20/200, multivariate analysis was then performed using 4 factors that were significant at *p* < 0.05 or less, or that were assumed to be involved. Fisher’s exact test was used to compare the incidence of complications between samples that were nonischemic or not, and between those that showed severe macular ischemia or not.

## 3. Results

Patients: Forty-four eyes of 44 patients were screened. Of these, 3 eyes were excluded because of the follow-up of less than 12 months. Finally, 41 eyes were enrolled. The patient characteristics are shown in Table 2. There were 27 males with 27 eyes and 14 females with 14 eyes. The mean age was 70.5 ± 12.2 years (range 46–94) and the mean follow-up period was 50.9 ± 21.6 months (range 19–93). The subtypes were nonischemic in 26 eyes, converted from nonischemic to ischemic in 9 eyes, and ischemic in 6 eyes. The mean ELM score, mean EZ score, and mean DRIL score were 1.12 ± 1.25, 1.12 ± 1.19, and 0.817 ± 0.960, respectively (Table 3). Twelve eyes had p-MLM, and 29 eyes did not. Regarding macular ischemia, 10 eyes had no macular ischemia (Grade 0) and 31 eyes had macula ischemia. Of the 31 eyes with macular ischemia, 18 eyes had mild macular ischemia (Grade 1), and 13 eyes had severe macular ischemia (Grade 2).

Treatments: The details are listed in Table 1. The average time from onset to first treatment was 1.26 ± 0.945 months (range 0.25–5.0). Intravitreal injection of anti-VEGF agents was performed in 34 eyes, IVA was performed in 31 eyes, IVR in 5 eyes, and 2 eyes had both IVA and IVR. The mean number of anti-VEGF agents’ injections at month 12 was 3.76 ± 2.63, and the mean number of injections during the follow-up period was 7.39 ± 6.67. Eleven eyes underwent STTA, two of which also underwent IVTA. PRP and vitrectomy were performed in 14 and 2 eyes, respectively.

**Figure 1:**
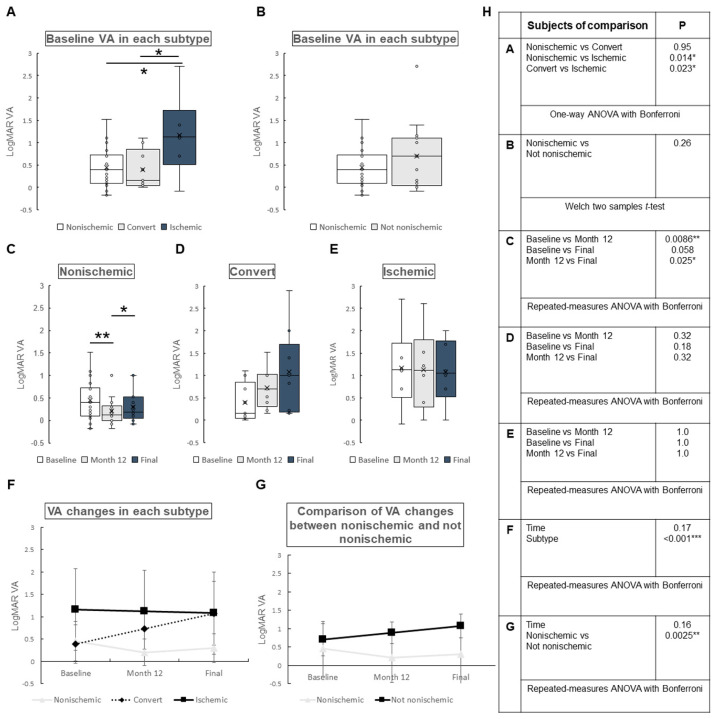
Change in logarithm of the minimal angle of resolution (LogMAR) visual acuity (VA) by subtypes of central retinal vein occlusion. (**A**) Baseline LogMAR VA in each subtype. Significant difference was found between nonischemic and ischemic types (*p* = 0.014 *), or convert (converted from nonischemic to ischemic) and ischemic types (*p* = 0.023 *). No significant difference was found between nonischemic type and convert type (*p* = 0.95). (**B**) A comparison of baseline LogMAR VA between nonischemic type and the others (not nonischemic). There was no significant difference between the groups (*p* = 0.26). (**C**–**E**) Changes in LogMAR VA in each subtype. (**C**) Nonischemic type. There was significant difference in LogMAR VA between baseline and month 12 (*p* = 0.0086), or month 12 and final (*p* = 0.025), and no significant difference between baseline and final (*p* = 0.058). (**D**) Convert type. There were no significant differences over time. (**E**) Ischemic type. There were no significant differences over time. (**F**,**G**) Comparison of LogMAR VA changes in each subtype (**F**) and between nonischemic or not (**G**). VA changes over time were not significantly different in each subtype (**F**) (*p* = 0.17) or between nonischemic and not nonischemic (**G**) (*p* = 0.16). In contrast, VA changes in each subtype (**F**) and between nonischemic and not nonischemic (**G**) were significantly different (*p* < 0.001, *p* = 0.0025, respectively). (**H**) Statistical methods used in (**A**) through (**G**) and the *p*-values. ANOVA = analysis of variance. * *p* < 0.05, ** *p* < 0.01, *** *p* < 0.001.

Visual acuity: The mean LogMAR VA for all patients was 0.544 ± 0.576, 0.456 ± 0.568, and 0.586 ± 0.665 at baseline, month 12, and final visit, respectively. No significant difference was found between baseline and month 12 (*p* = 0.77), baseline and final visit (*p* = 1.0), or month 12 and final visit (*p* = 0.12). The changes in LogMAR VA by subtypes are shown in Figure 1. At baseline, a significant difference in LogMAR VA was found between nonischemic type and ischemic type (*p* = 0.014), or converted type (converted from nonischemic to ischemic) and ischemic type (*p* = 0.023) (Figure 1A). No significant difference was found between types that were, or were not, nonischemic (*p* = 0.26) (Figure 1B). The changes in LogMAR VA for each subtype are shown in Figure 1C–E. Significant differences were found only in baseline vs. month 12 (*p* = 0.0086) and month 12 vs. final (*p* = 0.025) for the nonischemic type, but not for the other types (Figure 1C–E). LogMAR VA changes over time for each subtype are presented in Figure 1F,G. LogMAR VA changes were not significantly different over time (*p* = 0.17 in Figure 1F and *p* = 0.16 in Figure 1G) but were significantly different among the subtypes in Figure 1F (*p* < 0.001) and between nonischemic and not nonischemic in Figure 1G (*p* = 0.0025).

**Figure 2:**
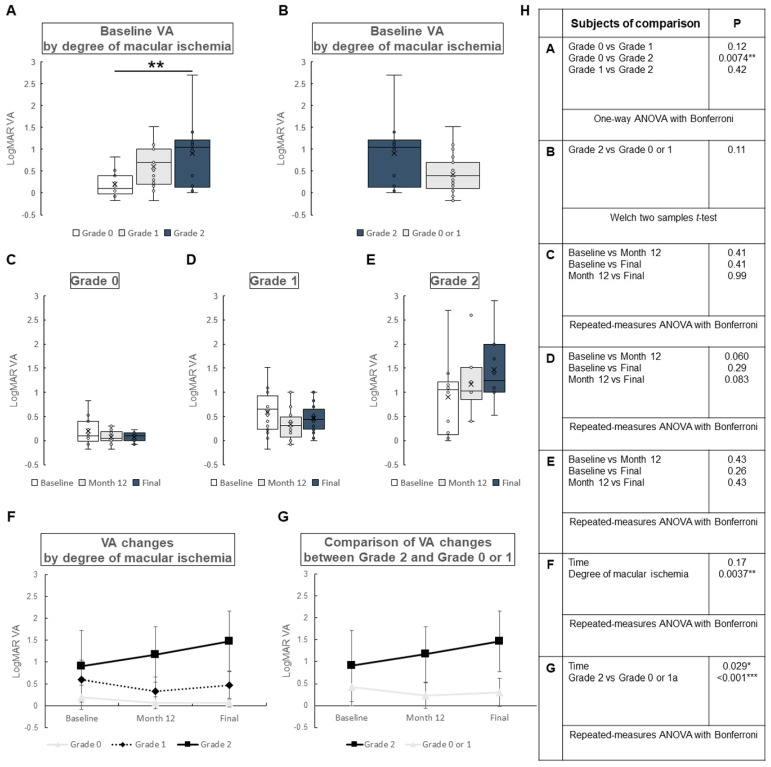
Change in logarithm of the minimal angle of resolution (LogMAR) visual acuity (VA) by degree of macular ischemia in central retinal vein occlusion. Grade 0: no macular ischemia, Grade 1: mild macular ischemia, Grade 2: severe macular ischemia. (**A**) Baseline LogMAR VA by degree of macular ischemia. There was significant difference in baseline LogMAR VA between those without macular ischemia (Grade 0) and those with severe macular ischemia (Grade 2) (*p* = 0.0074), but not between those without macular ischemia (Grade 0) and those with mild ischemia (Grade 1) (*p* = 0.12), or those with mild ischemia (Grade 1) and those with severe ischemia (Grade 2) (*p* = 0.42). (**B**) A comparison of baseline LogMAR VA between those with severe macular ischemia (Grade 2) and the others (Grade 0 or 1). There was no significant difference between the groups (*p* = 0.11). (**C**–**E**) Changes in LogMAR VA by degree of macular ischemia. (**C**) No macular ischemia group (Grade 0). There were no significant differences over time. (**D**) Mild macular ischemia group (Grade 1). There were no significant differences over time. (**E**) Severe macular ischemia group (Grade 2). There were no significant differences over time. (**F**,**G**) Comparison of LogMAR VA changes by degree of macular ischemia (**F**) and between severe macular ischemia (Grade 2) or not (Grade 0 or 1) (**G**). VA changes over time were not significantly different by degree of macular ischemia (**F**) (*p* = 0.17) but were significantly different between Grade 2 and Grade 0 or 1 (**G**) (*p* = 0.029). There was significant difference in VA changes by degree of macular ischemia (**F**) (*p* = 0.0037), or between Grade 2 and Grade 0 or 1 (**G**) (*p* < 0.001). (**H**) Statistical methods used in (**A**) through (**G**) and the *p*-values. ANOVA = analysis of variance. * *p* < 0.05, ** *p* < 0.01, *** *p* < 0.001.

The changes in LogMAR VA by degree of macular ischemia are shown in Figure 2. At baseline, significant difference was found between those without macular ischemia (Grade 0) and those with severe macular ischemia (Grade 2) (*p* = 0.0074) (Figure 2A). No significant difference was found between those without macular ischemia (Grade 0) and those with mild macular ischemia (Grade 1) (*p* = 0.12), or those with mild macular ischemia (Grade 1) and those with severe macular ischemia (Grade 2) (*p* = 0.42). No significant difference was found between those with severe macular ischemia (Grade 2) and those without severe macular ischemia (Grade 0 or 1) (*p* = 0.11) (Figure 2B). The changes in LogMAR VA by degree of macular ischemia are shown in Figure 2C–E. There were no significant differences among all subtypes. LogMAR VA changes over time for each subtype are presented in Figure 2F,G. LogMAR VA changes were not significantly different over time in Figure 2F (*p* = 0.17), but significant difference was found in Figure 2G (*p* = 0.029). The LogMAR VA changes over time were different by degree of macular ischemia (*p* = 0.0037), or between Grade 2 and Grade 0 or 1 (*p* < 0.001).

**Figure 3:**
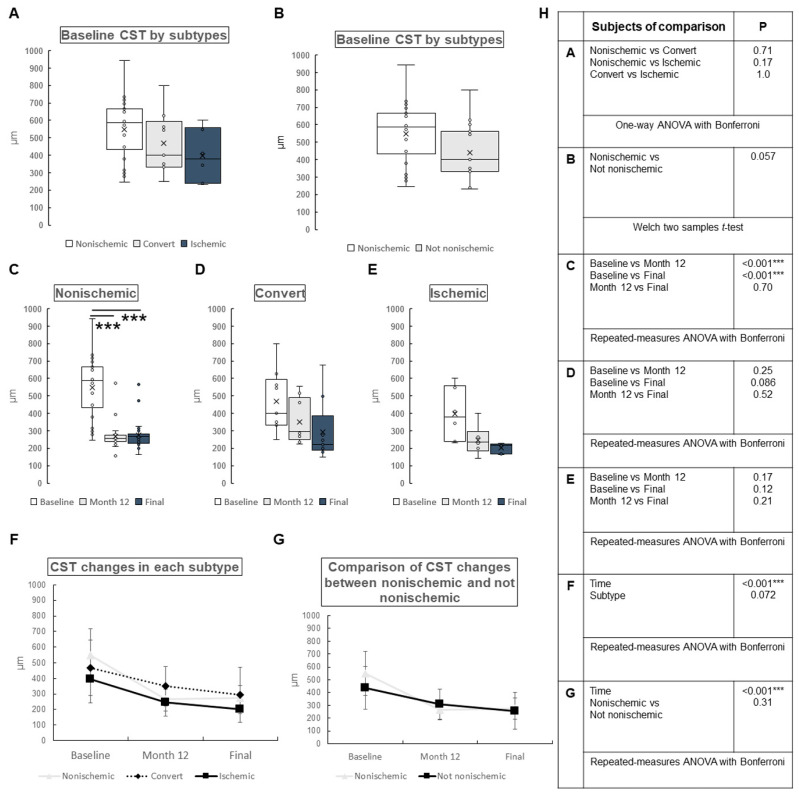
Change in central subfoveal thickness (CST) by subtypes of central retinal vein occlusion. (**A**) Baseline CST in each subtype. There were no significant differences among all subtypes. (**B**) A comparison of baseline CST between the nonischemic group and the others (not nonischemic). There was no significant difference between the groups (*p* = 0.057). (**C**–**E**) Changes in CST in each subtype. (**C**) Nonischemic group. There was significant difference in CST between baseline and month 12 (*p* < 0.001), or baseline and final (*p* < 0.001), and no significant difference between month 12 and final (*p* = 0.70). (**D**) Convert group. There were no significant differences over time. (**E**) Ischemic group. There were no significant differences over time. (**F**,**G**) Comparison of CST changes in each subtype (**F**) and between nonischemic and not nonischemic (**G**). The CST changes over time were significantly different in each subtype (**F**) (*p* < 0.001) and between nonischemic and not nonischemic (**G**) (*p* < 0.001). In contrast, CST changes in each subtype (**F**) or between nonischemic and not nonischemic (**G**) were not significantly different (*p* = 0.072 and *p* = 0.31, respectively). (**H**) Statistical methods used in (**A**) through (**G**) and the *p*-values. ANOVA = analysis of variance. *** *p* < 0.001.

CST: The mean CST for all patients was 507 ± 176, 282 ± 95.4, and 268 ± 107 µm at baseline, month 12, and final visit, respectively. Significant differences were found between baseline and month 12 (*p* < 0.001), and baseline and final (*p* < 0.001), but not between month 12 and final (*p* = 0.53). The changes of CST by subtype are shown in Figure 3. At baseline, there were no significant differences in CST among all of the subtypes (Figure 3A). Additionally, no significant difference was found between nonischemic and not nonischemic types (*p* = 0.057) (Figure 3B). The changes in CST for each subtype are shown in Figure 3C–E. Significant differences were found only in baseline vs. month 12 (*p* < 0.001) and baseline vs. final (*p* < 0.001) for the nonischemic type, but not for the other types (Figure 3C–E). CST changes over time for each subtype are presented in Figure 3F,G. CST changes were significantly different over time in Figure 3F (*p* < 0.001) and Figure 3G (*p* < 0.001), but were not significantly different by subtype in Figure 3F (*p* = 0.072) or between nonischemic and not nonischemic in Figure 3G (*p* = 0.31).

**Figure 4:**
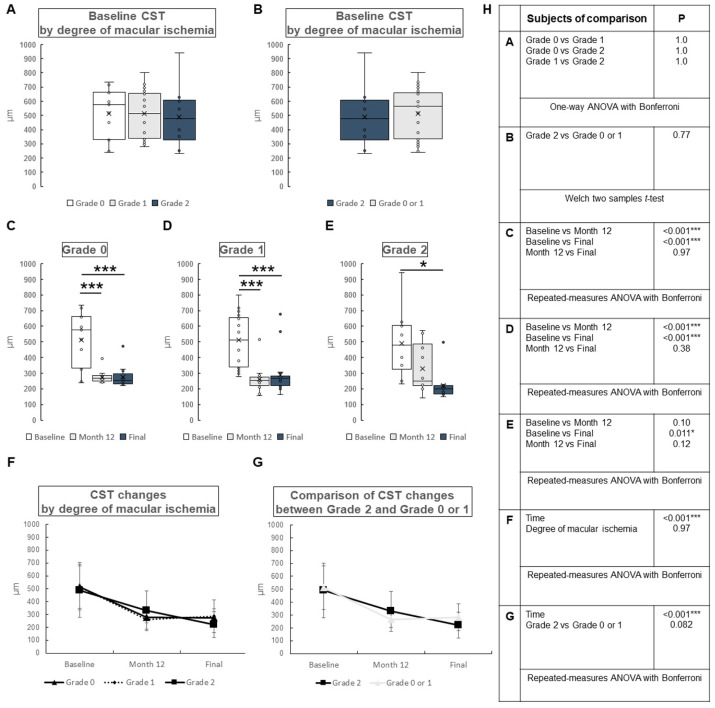
Change in central subfoveal thickness (CST) by degree of macular ischemia in central retinal vein occlusion. Grade 0: no macular ischemia, Grade 1: mild macular ischemia, Grade 2: severe macular ischemia. (**A**) Baseline CST by degree of macular ischemia. There were no significant differences among all groups. (**B**) A comparison of baseline CST between those with severe macular ischemia (Grade 2) and the others (Grade 0 or 1). There was no significant difference between the groups (*p* = 0.77). (**C**–**E**) Changes in CST by degree of macular ischemia. (**C**) No macular ischemia group (Grade 0). There was significant difference between baseline and month 12 (*p* < 0.001) and between baseline and final (*p* < 0.001). No significant difference was found between month 12 and final (*p* = 0.97). (**D**) Mild macular ischemia group (Grade 1). There was significant difference between baseline and month 12 (*p* < 0.001) and baseline and final (*p* < 0.001). No significant difference was found between month 12 and final (*p* = 0.38). (**E**) Severe macular ischemia group (Grade 2). There was significant difference between baseline and final (*p* = 0.011). Significant difference was not found between baseline and month 12 (*p* = 0.10), or between month 12 and final (*p* = 0.12). (**F**,**G**) Comparison of CST changes by degree of macular ischemia (**F**) and between Grade 2 and Grade 0 or 1 (**G**). CST changes over time were significantly different by degree of macular ischemia (**F**) (*p* < 0.001) and between Grade 2 and Grade 0 or 1 (**G**) (*p* < 0.001). In contrast, CST changes by degree of macular ischemia (**F**) or between Grade 2 and Grade 0 or 1 (**G**) were not significantly different (*p* = 0.97, *p* = 0.082, respectively). (**H**) Statistical methods used in (**A**) through (**G**) and the *p*-values. ANOVA = analysis of variance. * *p* < 0.05, *** *p* < 0.001.

The changes in CST by degree of macular ischemia are shown in Figure 4. At baseline, there were no significant differences among all groups (Figure 4A). There was no significant difference between those with severe macular ischemia (Grade 2) and those without severe macular ischemia (Grade 0 or 1) (*p* = 0.77) (Figure 4B). The changes in CST by degree of macular ischemia are shown in Figure 4C–E. In those without macular ischemia (Grade 0), significant differences were found between baseline and month 12 (*p* < 0.001) and baseline and final (*p* < 0.001), and no significant difference was found between month 12 and final (*p* = 0.97) (Figure 4C). In those with mild macular ischemia (Grade 1), significant differences were found between baseline and month 12 (*p* < 0.001) and baseline and final (*p* < 0.001), and no significant difference was found between month 12 and final (*p* = 0.38) (Figure 4D). In those with severe macular ischemia (Grade 2), significant difference was found between baseline and final (*p* = 0.011), and no significant differences were found between baseline and month 12 (*p* = 0.10), or for month 12 and final (*p* = 0.12) (Figure 4E). CST changes over time for each subtype are presented in Figure 4F,G. CST changes are shown to be significantly different over time in Figure 4F (*p* < 0.001) and in Figure 4G (*p* < 0.001), but no significant differences can be found by degree of macular ischemia in Figure 4F (*p* = 0.97), or between Grade 2 and Grade 0 or 1 in Figure 4G (*p* = 0.082).

Factors associated with the visual prognosis: Factors affecting the LogMAR VA at month 12 after treatment are shown in Table 4. In a univariate analysis, initial LogMAR VA (*p* < 0.001), subtype (ischemic: *p* = 0.0011), subtype (nonischemic: *p* < 0.001), ELM score (*p* < 0.001), EZ score (*p* < 0.001), DRIL score (*p* = 0.0033), presence of macular ischemia (Grade 1 or 2) (*p* = 0.0023), and presence of severe macular ischemia (Grade 2) (*p* < 0.001) are the significant factors. Multivariate analysis using initial LogMAR VA, ELM score, EZ score, and severe macular ischemia, which were significant factors in the univariate analysis, found that initial LogMAR VA (*p* < 0.001), ELM score (*p* = 0.030), and severe macular ischemia (Grade 2) (*p* < 0.001) are the significant factors.

Similarly, factors affecting the decimal visual acuity of 20/200 or less at final visit are shown in Table 5. In a univariate analysis, initial LogMAR VA (*p* = 0.043), initial CST (*p* = 0.046), subtype (ischemic: *p* = 0.046), subtype (nonischemic: *p* = 0.0025), ELM score (*p* < 0.001), EZ score (*p* = 0.0012), DRIL score (*p* = 0.048), presence of macular ischemia (Grade 1 or 2) (*p* = 0.048), and presence of severe macular ischemia (Grade 2) (*p* < 0.001) are the significant factors. Multivariate analysis using initial LogMAR VA, ELM score, EZ score, and severe macular ischemia, which were significant factors in the univariate analysis and were assumed to be involved, found that severe macular ischemia (Grade 2) is the only significant factor (*p* = 0.0092).

Complications: Serious complications were seen in 4 eyes out of 41 eyes (9.8%): one eye (2.4%) had VH, and 3 eyes (7.3%) had NVG (Table 6). The patient with VH had corrected-decimal visual acuity of 20/300 at initial visit and was ischemic with severe macular ischemia (Grade 2). PRP was performed at month 1 after the treatment, but the patient developed VH 46 months after the treatment. The VH spontaneously was resolved, and vitrectomy was not performed. The final corrected-decimal VA was 20/1000. Three eyes developed NVG. Two of the eyes had already developed NVG at the first visit, with decimal VA of hand motion and 20/250 at the first visit, respectively: both eyes were ischemic type with severe macular ischemia (Grade 2). Both underwent IVA and PRP, with final decimal VA of 20/2000 and 20/250. The other eye, which had a decimal VA of 20/250 at the first visit, converted from nonischemic to ischemic type at month 52 with severe macular ischemia (Grade 2), and ultimately became blind after IVA, vitrectomy, baerveldt glaucoma implant surgery, and ciliary laser.

The involvement of each subtype in the development of serious complications is shown in Table 7. Serious complications were significantly more common in the not-nonischemic type, i.e., the ischemic or convert types (*p* = 0.014 *). Additionally, those with severe macular ischemia (Grade 2) were significantly (*p* = 0.0071 **) more likely to develop serious complications.

**Figure 5:**
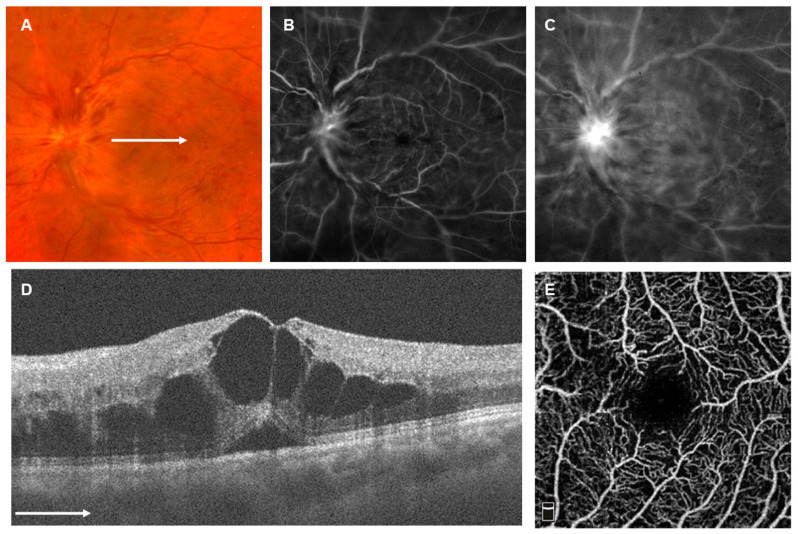
Representative case 1. Seventy-two-year-old male with central retinal vein occlusion in the left eye. (**A**) Fundus photograph (Optos California). Retinal veins were dilated and tortuous, with numerous retinal hemorrhages. (**B**,**C**) Fluorescein angiograms (Optos California). (**B**) Early phase and (**C**) late phase. Hyperfluorescence, possibly due to staining, was seen at the disc, and hyperfluorescence, possibly due to pooling, was seen at the macula. Nonperfusion areas were rarely seen and diagnosed as nonischemic. (**D**) Horizontal scan of optical coherence tomography (OCT) across the fovea (AVANTI OCT). Cystoid macular edema and serous retinal detachment were seen. (**E**) Superficial capillary layer of 3 × 3 mm OCT angiogram centered on the fovea (RTVue XR Avanti). The retinal vessel density of superficial capillary layer was 43.0% and determined to not be macular ischemia (Grade 0). The white arrows in (**A**,**D**) indicate which part of (**A**) was captured by the OCT image in (**D**).

Representative cases: Case 1: Seventy-two-year-old male developed CRVO in the left eye. The best-corrected decimal VA was 20/50 at the initial visit. OCT showed cystoid ME and serous retinal detachment (RD) with p-MLM; DRIL score, ELM score, and EZ score were all “0” (Figure 5). The eye was determined as a nonischemic type using FA images (Figure 5). STTA was performed for ME only once, and the ME spontaneously disappeared. The vessel density of the superficial capillary layer on the OCTA image within a 3 × 3 mm area centered on the fovea was 43.0% and determined there to be no macular ischemia (Grade 0) (Figure 5). The corrected VA was improved to 20/16 and was found to have remained there at the last observation at month 24.

**Figure 6:**
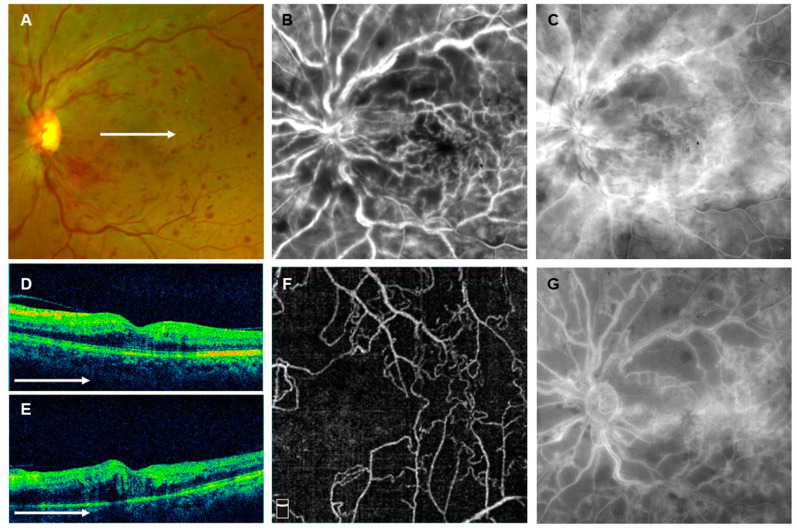
Representative case 2. Sixty-seven-year-old male with central retinal vein occlusion in the left eye. (**A**) Fundus photograph (Optos California). Retinal veins were dilated and tortuous, with numerous retinal hemorrhages. (**B**,**C**) Fluorescein angiograms (FAs) (Optos California). (**B**) Early phase and (**C**) late phase. Hyperfluorescence, possibly due to staining, was seen around the retinal veins. Small nonperfusion areas (NPAs) were seen in some places. (**D**,**E**) Horizontal scans of optical coherence tomography (OCT) across the fovea (Cirrus HD-OCT). (**D**) Initial visit and (**E**) two weeks later. Little or no macular edema (ME) was seen at the initial visit, but the ME was seen to have worsened two weeks later. (**F**) Superficial capillary layer of 3 × 3 mm OCT angiogram centered on the fovea (RTVue XR Avanti). The retinal vessel density of superficial capillary layer was 33.2% and determined to be severe macular ischemia (Grade 2). (**G**) Late phase of FA (Optos California). The NPAs were extensively enlarged, indicating a conversion to the ischemic type. The white arrows in (**A**,**D**,**E**) indicate which part of (**A**) was captured by the OCT image in (**D**,**E**).

Case 2: Sixty-seven-year-old male developed CRVO in the left eye. The best-corrected decimal VA was 20/20 at the initial visit. OCT showed ME, but no serous RD with p-MLM; DRIL score, ELM score, and EZ score were 1, 3, and 2, respectively (Figure 6). The eye was determined to be nonischemic using FA images at baseline but was converted to become ischemic at month 8 (Figure 6). The vessel density of superficial capillary layer on OCTA image within a 3 × 3 mm area centered on the fovea was 33.2% and was determined to be severe macular ischemia (Grade 2) (Figure 6). Six IVAs, one STTA, and one PRP were performed in the eye. The eye had no ME, but the best-corrected decimal VA was 20/200. Thereafter, there was no recurrence of ME, but the VA remained unchanged at the final follow-up of 45 months.

**Figure 7:**
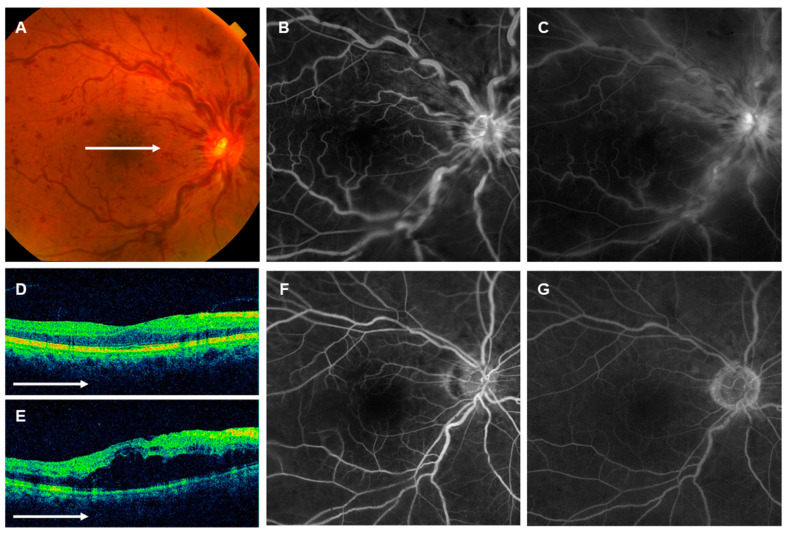
Representative case 3. Sixty-two-year-old male with central retinal vein occlusion in the right eye. (**A**) Fundus photograph (TRC-50AX). Retinal veins were dilated and tortuous, with numerous retinal hemorrhages. (**B**,**C**) Fluorescein angiograms (FAs) (Optos California). (**B**) Early phase and (**C**) late phase. Hyperfluorescence, possibly due to staining, was seen at the disc and around the retinal veins. (**D**,**E**) Horizontal scans of optical coherence tomography (OCT) across the fovea (Cirrus HD-OCT). (**D**) Initial visit and (**E**) two weeks later. Small macular edema (ME) was seen at the initial visit, but the ME was seen to have increased two weeks later. (**F**,**G**) FAs (Optos California) at month 4. (**F**) Early phase and (**G**) late phase. The hyperfluorescence seen at baseline had almost disappeared, but the macular area was hypofluorescent, with severe macular ischemia (Grade 2). The white arrows in (**A**,**D**,**E**) indicate which part of (**A**) was captured by the OCT image in (**D**,**E**).

Case 3: Sixty-two-year-old male developed CRVO in the right eye. The best-corrected VA was 20/20 at the initial visit, and ME was mild. The eye was determined to be nonischemic using FA images at baseline (Figure 7). The patient was followed up without any treatment because of their good VA, but after 2 weeks the best-corrected VA had decreased to 20/13. ME was exacerbated and three IVAs were performed. As a result, the ME disappeared, though the VA remained at 20/200 with no improvement. OCT images showed p-MLM with DRIL score, ELM score, and EZ score of 0, 1, and 0, respectively. The eye was determined via FA images to be suffering severe macular ischemia (Grade 2) (Figure 7).

## 4. Discussion

In this retrospective clinical study, we aimed to identify the factors influencing visual prognosis and the development of complications in eyes with CRVO. The findings, based on a cohort of 41 CRVO eyes that could be followed for more than one year, revealed no significant improvement in the LogMAR VA change, even when stratified by their status as ischemic or nonischemic, or by the extent of macular ischemia. Notably, significant differences were observed only in the nonischemic type between baseline and month 12 after treatment (Figure 1).

Conversely, the changes in CST were separately analyzed for each subtype, and for all subtypes, the mean CST exhibited a significant reduction at both year 1 and the final observation, when compared with the baseline. Despite this reduction in mean CST, the mean LogMAR VA did not improve.

Previous large clinical trials [6,7,8,9,10,11,12,13,14,15,16] that used anti-VEGF agents for eyes with ME associated with CRVO, have generally found significant reductions in CST and improvements in VA. However, these trials typically excluded patients with a decimal VA lower than 20/400 and those with severe ischemia. Consequently, the trials predominantly evaluated patients with relatively good vision or those anticipated to have a favorable visual prognosis.

Contrarily, Nagasato et al. [17] included cases with poor vision and severe ischemia into their evaluation, similarly revealing that, despite reductions in CST, there was no significant improvement in the mean VA, which aligns with the present findings and which more closely mirror real-world clinical practice. In actual clinical practice, some CRVO cases exhibit severe ischemia affecting the entire retina and/or macula, or cases in which the resolution of ME does not translate to significant vision improvement due to persistent macular ischemia. Unfortunately, effective treatment options for such cases at this stage remain limited.

Regarding the VA prognosis, the current study identified initial VA and severe macular ischemia as significant factors affecting VA at 12 months after treatment. Several previous reports have examined visual prognostic factors in eyes with CRVO. Most of these reports have highlighted the involvement of initial VA and age in visual prognosis [18,19,20]. Additionally, other reported factors include OCT biomarkers such as the DRIL score [23], p-MLM [24], ELM score [21], and EZ score [22]. Notably, the ELM and EZ scores, which represent the state of the outer retinal layers, appear to closely correlate with the visual prognosis.

Furthermore, Ghashut et al. [25] have investigated the relationship between the area of macular NPAs assessed by OCTA and VAs in eyes with CRVO. Based on these reports [18,19,20,21,22,23,24,25], the current study integrated the assessment of macular ischemia by OCTA into the prediction of VA prognostic factors, alongside initial VA, age, CRVO subtype, and various OCT biomarkers. Their findings underscore the way in which macular ischemia holds significant importance in the determination of visual prognosis. Additionally, Ghashut et al. [25] have explored factors influencing VA and retinal sensitivity in CRVO eyes, including age, VA and CST at the initial examination, paracentral foveal thickness, retinal sensitivity, EZ band defect length, and area of retinal NPAs in the macula. Their study reported an inverse relationship between the size of the retinal NPAs of the macula and VA as well as retinal sensitivity [25]. However, this analysis was univariate, not multivariate. Furthermore, the study did not analyze the subtypes of CRVO, the number of anti-VEGF injections, or the biomarkers on OCT such as DRIL, p-MLM, ELM, and EZ, which were assessed in the current study, as prognostic factors for VA outcomes.

In eyes with BRVO, the recurrence of ME is less likely when the macular ischemia is present [31,32], leading to a more favorable visual prognosis [31]. We agree with this observation in the case of BRVO, as the residual function of the non-occluded area often compensates for the severity of ischemia in the occluded area, contributing to an overly positive visual prognosis. Conversely, in eyes with CRVO, severe macular ischemia often extends to all quadrants surrounding the foveal avascular zone and leading to a generally poor visual prognosis, as there is no compensatory mechanism from the non-occlusive area (representative case 2). In addition, there are cases such as representative case 3, in which, despite being classified as nonischemic with mild macular ischemia across the entire retina, severe macular ischemia results in a poor visual prognosis. This insight sheds light on how severe macular ischemia can significantly impact the VA. In contrast, even in eyes with CRVO, if there is no macular ischemia or if the existing ischemia is mild, the visual prognosis can be relatively positive.

Dr. Nagasato et al. [17] have explored the baseline VA and systemic diseases as potential factors influencing the visual prognosis of eyes with CRVO. Their findings indicate that the baseline VA and the presence of internal carotid artery disease or diabetic retinopathy are associated with the visual prognosis [17]. In contrast, our current study primarily focused on local ocular biomarkers assessed by OCT and OCTA rather than systemic diseases.

Furthermore, in this study, we investigated the incidence of serious complications, particularly VH and NVG, in eyes with CRVO. VH was observed in one eye (2.4%), while NVG was identified in three eyes (7.3%) (Table 6). The occurrence of VH was noted in cases characterized by the ischemic type with severe macular ischemia. Among the three eyes with NVG, two were classified as ischemic and had already developed NVG at the initial visit, while the remaining eye had transitioned from nonischemic to ischemic. Notably, all three eyes were associated with severe macular ischemia. It is very interesting to observe that the presence or absence of severe macular ischemia also plays a crucial role in the development of such severe complications (Table 6 and Table 7). Prior research has suggested that the extent of posterior pole ischemia correlated with peripheral ischemia in eyes with diabetic retinopathy [33] or BRVO [34]. In cases of severe macular ischemia, the ischemic condition may extend to the entire fundus, potentially leading to complications such as VH and NVG. We have previously reported [31] that extensive posterior pole ischemia in eyes with BRVO is linked to the occurrence of NV or VH, which is consistent with the current study’s findings. However, the association with NVG was not analyzed in that study, due to its rarity in eyes with BRVO and the absence of actual NVG cases in the cohort [35]. For eyes with ischemic CRVO, laser treatment is often employed to prevent the development of retinal NV and NVG. In the current study, 15 eyes underwent laser treatment, comprising all 6 eyes of the ischemic type and all 9 eyes transitioning from the nonischemic to the ischemic type.

Although severe macular ischemia emerged as a critical determinant of visual prognosis, as exemplified in representative case 3, there were cases of poor visual prognosis attributed to severe macular ischemia, even within the nonischemic type. In such cases, the overall severity of retinal ischemia is comparatively milder, often without the development of retinal NV, VH, or NVG. Conversely, there were cases with a favorable visual prognosis despite the presence of mild macular ischemia, even within the ischemic type. Notably, the absence of observed retinal NV or NVG development in these cases could potentially be attributed to prior laser therapy due to the classification as an ischemic type.

The current study revealed that both the visual prognosis and complications were more intricately linked to the presence or absence of severe macular ischemia than to the traditional classification as ischemic or nonischemic. In the future, the presence or absence of macular ischemia may serve as a valuable criterion for assessing visual prognosis and complications in eyes with CRVO. Currently, anti-VEGF therapy for ME remains the mainstay of treatment in eyes with CRVO, with no established therapy targeting retinal ischemia. Developing a treatment approach targeting ischemia may be a potential consideration moving forward.

Several limitations are inherent in this study, including the limited number of cases, the single-institution focus, variations in treatment methods and strategies, the retrospective nature of the study, and the sole inclusion of Japanese patients, with potential racial differences remaining unknown. Future studies should aim to be prospective, multicentered, and encompass a large cohort for comprehensive analysis.

## Figures and Tables

**Table 1:** Grading of macular ischemia based on optical coherence tomography angiography (OCTA) vessel density. A 3 × 3 mm OCTA image centered on the fovea was taken, and the vascular density of the retinal superficial layer within that area was calculated.

Grade of Macular Ischemia	Definition
Grade 0 (No macular ischemia)	The vascular density of 40% or greater
Grade 1 (Mild macular ischemia)	The vascular density of 35% to 40%
Grade 2 (Severe macular ischemia)	The vascular density of 35% or less

**Table 2:** Demographics of the study population with central retinal vein occlusion.

	Total
Eyes, N	41
Gender, N (male/female)	27/14
Mean age, years (SD) (range)	70.5 (12.2) (46–94)
Mean follow-up, months (SD) (range)	50.9 (21.6) (19–93)
Mean baseline LogMAR visual acuity, (SD)	0.544 (0.576)
Mean central subfoveal thickness, μm (SD)	507 (176)
Diabetes, N (y/n)	9/32
Hypertension, N (y/n)	24/17
Subtype of CRVO, N (nonischemic/convert ^α^/ischemic)	26/9/6
Mean time from onset to first treatment, months (SD) (range)	1.26 (0.945) (0.25–5.0)
Mean number of injections of anti-VEGF agents within 12 months, N (SD)	3.76 (2.63)
Mean number of injections of anti-VEGF agents during the follow-up, N (SD)	7.39 (6.67)
STTA, N (y/n)	11/31
PRP, N (y/n)	14/27
Vitrectomy, N (y/n)	2/39

N = number, SD = standard deviation, LogMAR = logarithm of the minimal angle of resolution, y = yes, n = no, CRVO = central retinal vein occlusion, ^α^ converted from nonischemic to ischemic type, VEGF = vascular endothelial growth factor, STTA = sub-Tenon’s capsule injection of triamcinolone acetonide, PRP = pan-retinal photocoagulation.

**Table 3:** Biomarkers at month 12 on optical coherence tomography in the study population.

Biomarkers	Total
Mean ELM score, (SD)	1.12 (1.25)
Mean EZ score, (SD)	1.12 (1.19)
Mean DRIL score, (SD)	0.817 (0.960)
p-MLM, N (y/n)	12/29
Macular ischemia, N (Grade 0: none/Grade 1: mild/Grade 2: severe)	10/18/13

ELM = external limiting membrane, SD = standard deviation, EZ = ellipsoid zone, DRIL = disorganization of retinal inner layers, p-MLM = prominent middle limiting membrane, N = number, y = yes, n = no.

**Table 4:** Univariate and multivariate regression analysis for the detection of factors predictive of LogMAR visual acuity at month 12.

Univariate Regression Analysis			Multivariate Regression Analysis	
Factor	β	*p*	β (95% CI)	*p*
Age	0.0029	0.70		
Initial LogMAR VA	0.63	<0.001 ***	0.37 (0.19–0.56)	<0.001 ***
Initial CST	−0.00089	0.083		
Duration between symptom and initial therapy	−0.014	0.88		
Subtype (ischemic)	0.78	0.001 ***		
Subtype (nonischemic)	−0.68	<0.001 ***		
Number of injections of anti-VEGF agents within 12 months	−0.058	0.088		
ELM score at month 12	0.31	<0.001 ***	0.28 (0.030–0.54)	0.030 *
EZ score at month 12	0.30	<0.001 ***	−0.17 (−0.43–0.093)	0.20
DRIL score at month 12	0.27	0.0033 **		
Presence of p-MLM	0.19	0.34		
Presence of macular ischemia (Grade 1 or 2)	0.56	0.0023 **		
Presence of severe macular ischemia (Grade 2)	0.95	<0.001 ***	0.55 (0.28–0.83)	<0.001 ***

LogMAR = logarithm of the minimal angle of resolution, β = β coefficients, CI = confidence interval, VA = visual acuity, CST = central subfoveal thickness, VEGF = vascular endothelial growth factor, ELM = external limiting membrane, EZ = ellipsoid zone, DRIL = disorganization of retinal inner layers, p-MLM = prominent middle limiting membrane. * *p* < 0.05, ** *p* < 0.01, *** *p* < 0.001.

**Table 5:** Univariate and multivariate regression analysis for the detection of factors predictive of final decimal visual acuity of 20/200 or less.

Univariate Regression Analysis			Multivariate Regression Analysis	
Factor	OR (95% CI)	*p*	OR (95% CI)	*p*
Age	1.0 (0.95–1.1)	0.97		
Initial LogMAR VA	4.3 (1.1–18)	0.043 *	2.0 (0.13–29)	0.62
Initial CST	1.0 (0.99–1.0)	0.046 *		
Duration between symptom and initial therapy	0.54 (0.23–1.3)	0.16		
Subtype (ischemic)	6.8 (1.0–44)	0.046 *		
Subtype (nonischemic)	0.087 (0.018–0.43)	0.0025 **		
Number of injections of anti-VEGF agents within 12 months	0.76 (0.57–1.0)	0.074		
ELM score at month 12	4.0 (1.8–8.8)	<0.001 ***	1.8 (0.10–30)	0.70
EZ score at month 12	4.4 (1.8–11)	0.0012 **	1.5 (0.068–32)	0.80
DRIL score at month 12	2.1 (1.0–4.3)	0.048 *		
Presence of p-MLM	2.2 (0.54–9.4)	0.27		
Presence of macular ischemia (Grade 1 or 2)	0.11 (0.013–0.98)	0.048 *		
Presence of severe macular ischemia (Grade 2)	85 (7.7–910)	<0.001 ***	31 (2.3–410)	0.0092 **

OR = odds ratio, CI = confidence interval, LogMAR = logarithm of the minimal angle of resolution, VA = visual acuity, CST = central subfoveal thickness, VEGF = vascular endothelial growth factor, ELM = external limiting membrane, EZ = ellipsoid zone, DRIL = disorganization of retinal inner layers, p-MLM = prominent-middle limiting membrane. * *p* < 0.05, ** *p* < 0.01, *** *p* < 0.001.

**Table 6:** Serious complications in the study population.

Complications	Number of Eyes	Initial Decimal Visual Acuity	Subtype	Macular Ischemia	Time from Initial Visit to Onset of Complications, Months	Treatments	Final Decimal Visual Acuity
VH	1 eye	20/300	Ischemic	Severe (Grade 2)	46	STTA + PRP	20/1000
NVG	3 eyes	Hand motion	Ischemic	Severe (Grade 2)	Already developed at the initial visit	IVA + PRP	20/2000
		20/500	Ischemic	Severe (Grade 2)	Already developed at the initial visit	IVA + PRP	20/250
		20/250	Convert ^α^	Severe (Grade 2)	52	IVA	No light perception
						Vitrectomy + PRP + BGI	
						Ciliary laser	

VH = vitreous hemorrhage, STTA = sub-Tenon’s capsule injection of triamcinolone acetonide, PRP = pan-retinal photocoagulation, NVG = neovascular glaucoma, IVA = intravitreal injection of aflibercept, ^α^ converted from nonischemic to ischemic type, BGI = baerveldt glaucoma implant.

**Table 7:** Comparison of incidence of serious complications in the study population.

	Presence of Serious Complications		*p*
	Yes, N	No, N	
Ischemic and convert ^α^	4	11	0.014 *
Nonischemic	0	26	
Grade 2 macular ischemia	4	9	0.0071 **
Grade 0 or 1 macular ischemia	0	28	

N = number. ^α^ converted from nonischemic to ischemic type. Fisher’s exact test. * *p* < 0.05, ** *p* < 0.01.

## Data Availability

The datasets used and/or analyzed during the current study are available from the corresponding author on reasonable request.
